# A Randomized, Controlled Trial Exploring Collaborative Nursing Intervention on Self-Care Ability and Blood Glucose of Patients with Type 2 Diabetes Mellitus

**DOI:** 10.1155/2022/7829454

**Published:** 2022-03-22

**Authors:** Xi Wang, Jin Liang, Wei Yang

**Affiliations:** ^1^Department of Gynecology, China-Japan Union Hospital of Jilin University, Changchun, China; ^2^Department of Gastrointestinal and Colorectal Surgery, China-Japan Union Hospital of Jilin University, Changchun, China; ^3^Department of Hepatopancreatobiliary Surgery, China-Japan Union Hospital of Jilin University, Changchun, China

## Abstract

**Objective:**

For determining the impacts of collaborative nursing intervention (CNI) on self-care ability and blood glucose (BG) of patients with type 2 diabetes mellitus (T2DM).

**Methods:**

The study enrolled 72 T2DM patients, who are referred to our hospital between April 2017 and September 2019. Of them, 35 cases given routine nursing were set as the control group (CG) and 37 cases given CNI were set as the research group (RG). The Exercise of Self-Care Agency (ESCA) scale scores and the levels of fasting plasma glucose (FPG) as well as glycosylated hemoglobin (HbAlc) were observed pre- and postintervention. The scores of SAS and HAMD and Morisky pre- and postnursing intervention as well as postnursing SF-36 scores and patients' satisfaction toward the nursing content were recorded.

**Results:**

After intervention, RG presented notably lower serum HbAlc and FPG levels than CG (*P* < 0.05); RG presented evidently lower SAS and HAMD scores while distinctly higher Morisky, SF-36, and ESCA scores than CG (*P* < 0.05); the nursing satisfaction in RG and CG was 97.30% and 51.43%, respectively.

**Conclusions:**

In view of the fact that CNI can decrease HbAlc and FPG levels in patients with T2DM and enhance their self-care ability, it is worth popularizing in the clinic.

## 1. Introduction

Diabetes mellitus (DM) is a ubiquitous metabolic dysregulation with a terribly high incidence across the globe [1]. It is a set of metabolic diseases featured with hyperglycemia due to insulin secretion deficiency or insulin action or the two. Chronic hyperglycemia of diabetes is bound up with long-run injury, dysfunction, and organ failure, especially the nerves, kidneys, eyes, and heart as well as blood vessels [2]. DM is currently the illness with the highest incidence worldwide, and society advancement and improvement of people's living standards are driving the increasing incidence of DM [3]. According to research statistics, the proportion of diabetes worldwide reached 25.6% in 2015 [4]. DM can predispose people to complications like nervous system diseases and kidney diseases. Once the disease deteriorates because of the absence of timely therapy, it will lead to malignant tumours directly. DM, defined by elevated blood glucose (BG) markers, is a primary risk factor for cardiovascular illnesses, which bears the major responsibility for death in diabetic patients [5]. The treatment of diabetes is still a challenge. Clinically, efforts have been made to find a way to effectively prevent and treat diabetes, but no significant breakthrough has been made so far [6]. Hence, early screening and diagnosis are of utmost importance.

Patients with DM need long-time medication, and some also require insulin injections to control their BG. And during treatment, patients' compliance and awareness of the disease directly affect their BG status and mental health. Today, the major obstacle that stands in the way of nursing work is how to make patients face diabetes actively and rationally and receive professional and systematic treatment [7–9]. The concept of collaborative nursing intervention (CNI) mode is to give full play to patients' self-care ability on the basis of accountability nursing, encourage patients and their families to take part in the process of health care, maximize patients' subjective initiative and treatment enthusiasm, and creatively utilize existing manpower and material resources [10, 11]. Such a nursing model has been applied to the care of patients with cardiovascular surgery, AIDS, depression, and schizophrenia [12][13][14], but whether it can play a role in diabetes has not been indicated. In light of this, we investigate the impacts of CNI on self-care ability and BG of type 2 diabetes mellitus (T2DM) patients, with the aim of rendering evidence and advice for future clinical practice.

## 2. Materials and Methods

### 2.1. General Information

This was a randomized, controlled trial. Totally, 72 T2DM patients admitted to our hospital between Apr. l, 2017, and Sep. 2019 were enrolled as the study population by using the continuous fixed-point sampling method, of whom 35 patients in the control group (CG) received routine nursing, and 37 patients in the research group (RG) were given CNI. The hospital Medical Ethics Committee approved the study protocol without reserves with the license no. of 2016.239.22.

### 2.2. Inclusion and Exclusion Criteria

Inclusion criteria include (1) patients diagnosed with diabetes according to the diagnostic guidelines for diabetes issued in 2014 [15] and treated in our hospital, (2) patients with detailed case data, (3) patients glad to cooperate and take part in the study, (4) patients between 30 and 65 years old, (5) patients without other severe organ diseases impacting this study, and (6) patients who provided informed consent signed by the patient himself/herself or his/her next of kin.

Exclusion criteria include (1) patients who died in the process of therapy, (2) patients comorbid with other tumours or other cardiac-cerebral vascular illnesses, (3) patients with physical disability, (4) pregnant patients, (5) patients comorbid with other autoimmune illnesses or other chronic illnesses, (6) referred patients, and (7) patients with mental disorders, speech disorders, or diseases that affect the results of this study.

### 2.3. Nursing Methods

CG received routine nursing care: diet nursing: nurses gave health education to patients and advised them to eat multiple small meals on a regular basis and follow the dietary principles of low sugar and fat, proper protein, and high fiber and vitamins; appropriate exercise: appropriate exercise was conducted to intensify the body immunity, elevate the sensitivity to insulin, and help patients keep BG under control. In addition, the nurses instructed the patients to positively take part in aerobic activities like jogging and yoga.

RG adopted cooperative nursing: RG implemented CNI based on routine nursing from the following dimensions: knowledge guidance: manuals regarding health education were distributed to patients and their families, and diabetes-related knowledge such as the effects and adverse reactions of common drugs for diabetes was explained to them, together with the guidance on daily dietary and lifestyle. Besides, the contents of electrolyte, BG, and glycosylated hemoglobin (HbAlc) were reviewed regularly by following the doctor's suggestions, and patients were guided to read examination results such as BG and blood lipid. Self-care strengthening: the responsible nurse explained diabetes knowledge to patients and their families, provided psychological counseling, and encouraged patients to actively participate in the nursing diagnosis and treatment process, so that patients can control BG at the ideal level through self-care and self-psychological adjustment. What is more, medical professionals set up files for patients, developed care plans according to the patient's condition, and hired diabetes experts to conduct learning concerning exercise, diet, and disease-related knowledge every week. The responsible nurse maintained telephone contact with patients and conducted weekly telephone follow-ups to collect and analyze patient information. Moreover, questionnaires were distributed and retrieved by specialized nursing staff on admission and one month after discharge. Psychological nursing: the responsible nurse listened to the patient's complaints carefully and patiently, coordinated with the family to give care and support to patients, and conducted psychological nursing according to the patient's individual psychological characteristics. Besides, the nurses try their best to relieve patients' psychological pressure and helped them to cope with various psychological problems in the treatment of the disease and to enhance their adaptability. In particular, for patients with excessive pressure and mood swings during hospitalization, the responsible nurse provided patients with certain psychological counseling and support and explained to patients the great role of a good state of mind and mood in conquering the disease and promoting rehabilitation, hoping that patients can cooperate with the treatment with a pleasant and relaxed attitude.

### 2.4. Scoring Criteria

Self-Rating Anxiety Scale 9.4 software (SAS Institute, Inc., USA) and Hamilton Depression Rating Scale (HAMD; Hamilton, 1960) were employed for mental health assessment. The SAS has a full score of 100 points with mild anxiety corresponding to a SAS value 50-70, moderate anxiety to a SAS value 71-90, and severe anxiety to a SAS value > 90. HAMD composed of 24 items evaluated patients' depression. The score was positively bound up with depression severity.

The Morisky medication adherence scale (MMAS; 2006 Donald E. Morisky) [16] was utilized to evaluate the therapy compliance of patients pre- and postnursing intervention from four respects, namely, diet control, following the doctor's advice, body mass control, and proper exercise. With a full score of 50 points, full compliance corresponded to a MMAS value of 50, partial compliance to a MMAS value between 30 and 40 points, and noncompliance to a MMAS value of less than 30 points. Self-care ability was assessed via the Exercise of Self-Care Agency (ESCA, Hanson and Bickel's 1985), which consists of 4 dimensions and 43 items, with a full score of 172 points. A higher score denotes better self-care ability. The quality of life (QOL) of patients was assessed via the SF-36, which covered physical health (role-physical, somatic pain, overall health, and physiological function) and mental health (social function, vitality, emotional role, and mental health), with 8 dimensions, each corresponding to 100 points. A higher score denotes better QOL of the patient. The nursing satisfaction questionnaire (20 questions, 5 points each) developed by our hospital was adopted for scoring the patients' satisfaction toward nursing, with questionnaire Cronbach's alpha representing internal consistency of criteria *A* 0.93: a total score < 70: dissatisfaction; 70-89: satisfaction; ≥90: high satisfaction: Satisfaction = (high satisfaction + satisfaction)cases/the sum of cases × 100%.

### 2.5. Blood Sampling and Main Reagents

Morning fasting venous peripheral blood (5 mL) was placed at room temperature for 30 min before centrifuging for 10 min (3000 rpm/min), and the resulting upper serum was subpackaged into enzyme-free EP tubes. Part of the serum samples was processed for experiment, while the rest were kept at -80°C until use. Following the manufacturer's instructions, the determination of BG function (glycosylated hemoglobin: HbAlc, fasting plasma glucose: FPG) was made with an automatic biochemical analyzer (Jiaozuo Lufeifan Biotechnology Co., Ltd., Cat. No. LFF-LC-1781). An Eppendorf CryoCube F740hi ultralow temperature refrigerator was obtained from Eppendorf Ltd., China (Cat. No. ep000000).

### 2.6. Outcome Measures

Primary endpoints: the ESCA scores and serum HbAlc and FPG levels pre- and postnursing intervention were observed. The patients' venous blood was measured at 0 weeks and after treatment.

The scores of SAS, HAMD, and Morisky pre- and postnursing intervention as well as postnursing SF-36 scores and patients' satisfaction toward nursing were recorded.

### 2.7. Statistical Analyses

This study statistically analyzed the obtained data via SPSS20.0 (IBM Corp, Armonk, NY, the States) and visualized them via GraphPad 7. The K-S test was adopted for analyzing the distribution of the measurement data, among which those in normal distribution were presented by mean ± SD. The independent sample *t*-test and paired *t*-test were utilized for intragroup comparisons and intergroup comparisons, respectively. The counting data (%) were subjected to analysis by the chi-squared test (denoted by *χ*^2^). *P* < 0.05 denotes a notable difference.

## 3. Results

### 3.1. Clinical Data

The two cohorts differed insignificantly in terms of smoking history, age, BMI, residence, dietary preference, drinking history, exercise habits, HbAlc, and FPG, which was suggestive of comparability (*P* > 0.05) (Table 1).

### 3.2. HbAlc and FPG Levels Pre- and Postintervention

**Figure 1:**
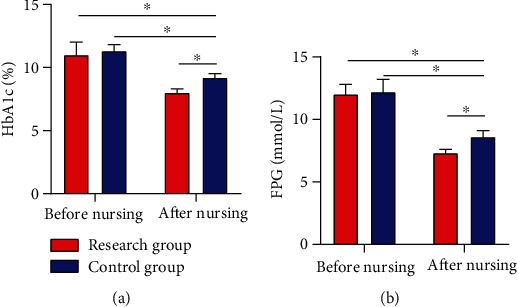
HbAlc and FPG levels pre- and postintervention. (a) After intervention, the HbAlc expression in the RG dropped notably and was lower than that in the CG. (b) After intervention, the FPG expression in the RG dropped notably and was lower than that in the CG. Note: ^∗^*P* < 0.05 between the two groups.

Evident differences were absent regarding serum HbAlc (9.95 ± 1.00) and FPG (9.92 ± 2.13) between the two cohorts before intervention (*P* > 0.05); however, lower HbAlc (7.45 ± 0.45) and FPG (6.95 ± 1.50) levels were observed in RG after it (*P* < 0.05) (Figure 1).

### 3.3. SAS Scores Pre- and Postintervention

**Figure 2:**
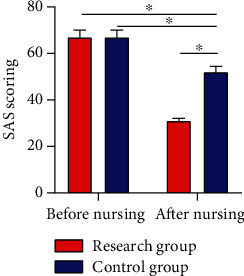
SAS scores pre- and postintervention. After intervention, the SAS score in the RG dropped notably and was lower than that in the CG. Note: ^∗^*P* < 0.05 between the two groups.

No difference was noted between the two cohorts in the SAS score before intervention (*P* > 0.05); however, a more evident decrease in the SAS score was observed in RG after it (*P* < 0.05) (Figure 2).

### 3.4. HAMD Scores Pre- and Postnursing Intervention

**Figure 3:**
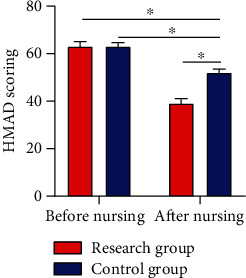
HAMD scores pre- and postintervention. After intervention, the HAMD score in the RG dropped notably and was lower than that in the CG. Note: ^∗^*P* < 0.05 between the two groups.

The two cohorts were similar in the HAMD score before intervention (*P* > 0.05), but after it, the HAMD score was significantly lower in RG than in CG (*P* < 0.05) (Figure 3).

### 3.5. Morisky Scores Pre- and Postnursing Intervention

**Figure 4:**
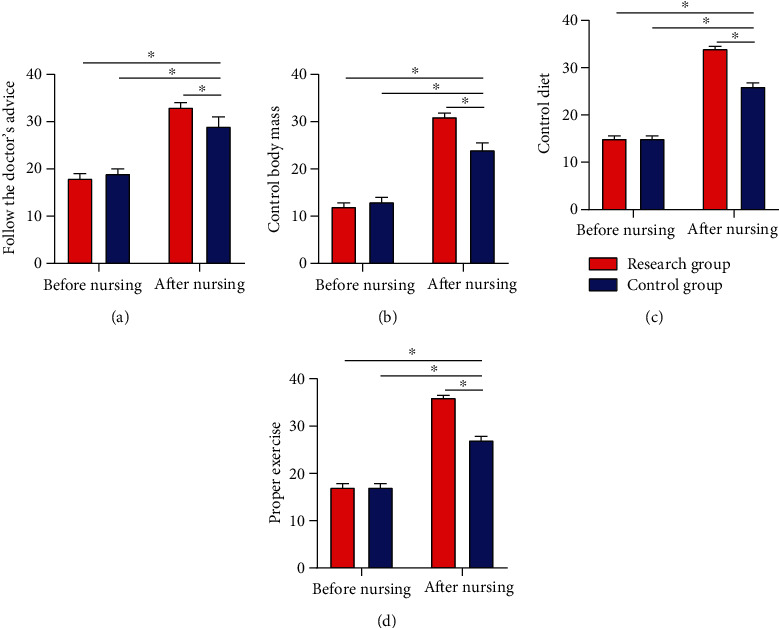
Morisky scores pre- and postintervention. (a) After intervention, the RG got a notably higher score of following the doctor's advice than the CG. (b) After intervention, the RG got a notably higher score of body mass control than the CG. (c) After intervention, the RG got notably higher scores of diet control than the control. (d) After intervention, the RG got a notably higher score of proper exercise than the CG. Note: ^∗^*P* < 0.05 between the two groups.

The Morisky score showed no notable difference between the two cohorts before intervention (*P* > 0.05). After it, RG showed a notably higher Morisky score than CG in the following four aspects: body mass control, following the doctor's advice, proper exercise, and diet control (*P* < 0.05) (Figure 4).

### 3.6. SF-36 Scores after Nursing Intervention

**Figure 5:**
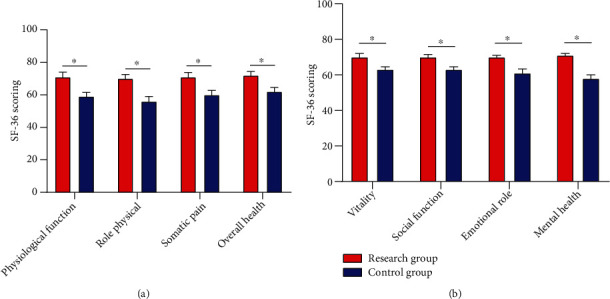
SF-36 scores postintervention. (a) The RG got notably higher physical health scores of SF-36 than the CG. (b) The RG got notably higher mental health scores of SF-36 than the CG. Note: ^∗^*P* < 0.05 between the two groups.

Observation of the SF-36 scores in the two groups revealed notably better physical health (physiological function, role-physical, somatic pain, and overall health) and mental health (vitality, social function, emotional role, and mental health) in RG than in CG (*P* < 0.05) (Figure 5).

### 3.7. ESCA Scores Pre- and Postnursing Intervention

**Figure 6:**
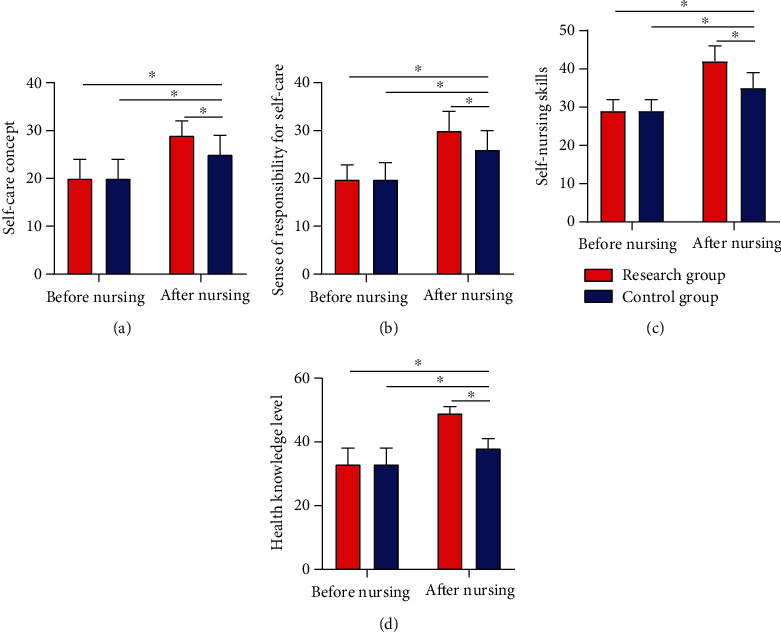
ESCA scores pre- and postintervention. (a) After intervention, the RG got notably higher scores of self-care concept than the CG. (b) The RG got notably higher scores of sense of responsibility for self-care than the CG after intervention. (c) The RG got notably higher scores of self-nursing skills than the CG after intervention. (d) The RG got notably higher scores of health knowledge level than the CG after nursing intervention. Note: ^∗^*P* < 0.05 between the two groups.

Observation of ESCA scores before and after intervention determined no evident difference between the two cohorts before it (*P* > 0.05), while observably higher scores of self-care concept, sense of responsibility for self-care, self-nursing skills, and health knowledge level in RG than in CG postnursing intervention (*P* < 0.05) (Figure 6).

### 3.8. Patient Satisfaction with Nursing Content

The nursing satisfaction of patients in RG was 97.30%, evidently higher than 51.43% in CG (*P* < 0.05) (Table 2).

## 4. Discussion

DM is a group of metabolic diseases featured with hyperglycemia due to insulin secretion deficiency/insulin action or the two [17]. In 2014, the prevalence of diabetes was estimated to be 9% worldwide [18], and approximately 1.6 million people died of the illness worldwide in 2015 [19]. The disease is also bound up with high morbidity because of extensive complications like nephropathy, retinopathy, neuropathy, and cardiovascular diseases [20, 21]; thus, preventing and managing these complications have become the primary aspect of modern diabetes care. The CNI model emphasizes that nurses, patients, and family members are integrated into nursing work, so that patients and family members can gradually learn and master the condition monitoring and nursing skills during diagnosis and treatment, which is the best nursing model to improve the QOL of patients with T2DM [22, 23].

In our study, no notable difference was observed in HbAlc and FPG levels between the two cohorts before nursing intervention, while the two parameters were notably lower in RG than in CG after intervention, which indicated that CNI could effectively control the BG level of patients. Under normal circumstances, nervous and excited mood and psychological pressure will stimulate the substantial secretion of stress hormones that are antagonistic to insulin, such as adrenocortical hormone, glucagon, and norepinephrine, making it more difficult for diabetic patients to control BG. Studies have shown that [24], compared with the nondiabetic population, people with T2DM are more susceptible to subclinical and clinical symptoms of anxiety. Traditionally, anxiety has been related to unfavorable metabolic outcomes and elevated medical complications in T2DM patients and has an adverse impact on their self-awareness of health and QOL. By observing the Morisky and ESCA scores of patients in the two cohorts, we found that the treatment compliance and self-care ability improved notably after intervention, with better parameters in RG, suggesting that CNI could effectively improve patients' self-management ability and treatment compliance. The CNI model has been used for multidisciplinary therapy of mental health problems and chronic diseases and has been proved to be successful in managing the pathology of depression, which accompanies diabetes in most cases [25]. With the transformation of modern medical mode and the raise of people's health awareness, treatment is to improve and prolong the survival of patients and also to improve their QOL. CNI is a novel nursing mode, which emphasizes nurses as supporters and educators in the medical sector and deeply reflects the crucial part of patients' involvement in nursing work. In addition to accountability nursing, CNI also covers other dimensions like psychological nursing and health education, aimed at encouraging patients to take part in nursing work and clinical therapy, giving enough play to patients' self-care ability, and improving their enthusiasm as well as initiative in treatment [26, 27]. Lastly, we used the self-made nursing satisfaction questionnaire of our hospital for evaluating patient satisfaction toward nursing and found 97.30% and 51.43% of nursing satisfaction in RG and CG, respectively. The results indicate that CNI is unanimously appreciated by the patients and their families, which proved the practicability of CNI and its enormous success in clinical practice future.

Based on the above research, we preliminarily proved the ability of CNI in validly controlling the BG level of patients with diabetes and improving their self-care ability. However, there are still some deficiencies. First, this study only adopts routine nursing as a control instead of other care models out there, which is relatively single. Second, patient follow-up should be supplemented in the future research design. All in all, more nursing models will be included as controls, and prognostic follow-up of patients will be added in future studies, so as to supplement the comprehensiveness of our research and support our research results.

To sum up, given that the CNI model can strongly boost the self-care ability of T2DM patients, effectively control the BG level, and improve their treatment compliance, it is worth popularizing in clinical nursing of T2DM.

## Figures and Tables

**Table 1:** Basic data (*n* (%)).

	RG (*n* = 37)	CG (*n* = 35)	*χ* ^2^ or *t*	*P*
Age (years old)	42.4 ± 9.6	42.2 ± 10.2	0.086	0.932
BMI	23.05 ± 1.24	23.02 ± 1.17	0.106	0.916
History of smoking			1.167	0.683
Yes	9 (24.32)	10 (28.57)		
No	28 (75.68)	25 (71.43)		
History of drinking			0.500	0.479
Yes	17 (45.95)	19 (54.29)		
No	20 (54.05)	16 (45.71)		
Residence			0.052	0.820
Urban	31 (83.78)	30 (85.71)		
Rural	6 (16.22)	5 (14.29)		
Dietary preference			0.481	0.488
Light	7 (18.92)	9 (25.71)		
Spicy	30 (81.08)	26 (74.29)		
Exercise habits			0.445	0.505
Yes	11 (29.73)	13 (37.14)		
No	26 (70.27)	22 (62.86)		
HbAlc (%)	11.03 ± 1.18	11.05 ± 1.12	0.074	0.942
FPG (mmol/L)	11.54 ± 1.28	11.51 ± 1.23	0.101	0.920

**Table 2:** Nursing satisfaction scores (*n* (%)).

Groups	Number of cases	Satisfied	Relatively satisfied	Dissatisfied	Satisfaction (%)
RG	37	30 (81.08)	6 (16.22)	1 (2.70)	36 (97.30)
CG	35	8 (22.86)	10 (28.57)	17 (48.57)	18 (51.43)
t	—	—	—	—	20.180
P	—	—	—	—	0.001

## Data Availability

The datasets used during the present study are available from the corresponding author upon reasonable request.
